# Single Versus Shared ICU Rooms and the Risk of Unplanned Extubation: A Real-World Cohort Showing Tube Displacement as an Early Signal

**DOI:** 10.7759/cureus.102722

**Published:** 2026-01-31

**Authors:** Beatriz Amaral Costa Savino, Danilo Franco Guidi, Silvia Helena Ferraz Planard, Viviane Perin, Bruno Augusto Goulart Campos

**Affiliations:** 1 Department of Critical Care Medicine, Hospital de Clínicas, University of Campinas (UNICAMP), Campinas, BRA

**Keywords:** airway management, extubation, intensive care units, mechanical ventilation, patient safety

## Abstract

Background: Unplanned extubation (UE) is a critical adverse event in intensive care units (ICUs) that can result in severe complications and increased mortality. Environmental factors, such as room configuration, may influence this risk but remain understudied. This study aimed to evaluate the association between ICU room type (single vs. shared) and the occurrence of UE in mechanically ventilated patients.

Methods: This was a retrospective cohort study conducted at the Hospital de Clínicas, University of Campinas, Brazil, including 118 adult patients admitted between March 2023 and March 2024. Variables analyzed included age, sex, Simplified Acute Physiology Score 3 (SAPS 3) score, room type, extubation type (planned vs. unplanned), tube displacement, extubation failure (reintubation ≤48 hours), hospital mortality, ICU mortality, and ventilator-associated pneumonia. Statistical analyses used chi-square, Fisher’s exact, Student’s t, and Mann-Whitney U tests, with a significance level of p < 0.05.

Results: Of 118 patients, 54.3% were men, with a mean age of 56.8 years and a mean SAPS 3 score of 76.4. Forty-five patients (38.1%) were admitted to single rooms and 73 (61.9%) to shared rooms. UE occurred in 13.6% of cases and was significantly more frequent in single rooms (p = 0.031). Tube displacement was associated with UE (p = 0.016), and extubation failure correlated with increased hospital (p = 0.006) and ICU mortality (p = 0.019).

Conclusions: Room type influenced extubation patterns but did not affect major clinical outcomes. Tube displacement was identified as an early marker for UE risk, emphasizing the need for continuous monitoring and preventive strategies.

## Introduction

Unplanned extubation (UE) is one of the most critical adverse events in intensive care units (ICUs) [1]. It includes self-extubation, when the patient removes the endotracheal tube, and accidental extubation resulting from unintended movements or handling errors by healthcare staff [2]. Its incidence ranges from 1% to 3% in ICUs but may be higher in settings characterized by increased patient turnover, higher workload, or suboptimal surveillance processes [3].

UE is associated with significant morbidity, including upper airway injury, aspiration, hypoxemia, and increased risk of mortality, frequently requiring emergency reintubation and additional intensive therapy [4,5]. Identification of factors associated with UE is therefore essential for prevention. Previously reported contributors include patient-related characteristics, depth of sedation, comorbidities, and aspects of nursing care and monitoring [6].

Beyond patient- and care-related factors, the physical ICU environment may also influence the risk of UE, although this aspect remains less explored. ICU room configuration directly affects visibility, proximity between staff and patients, and the dynamics of continuous surveillance [7]. Single-patient rooms may provide benefits such as increased privacy and reduced environmental stress; however, they may also reduce direct line-of-sight monitoring, potentially delaying recognition of early warning signs such as agitation or endotracheal tube displacement. In contrast, shared rooms facilitate continuous visual observation but may be associated with increased noise, interruptions, and staff workload [8].

Despite growing interest in ICU design, evidence on how architectural configuration influences the occurrence of UE remains limited and inconsistent [9]. Understanding whether and how room type affects extubation safety has direct clinical relevance, as ICU architectural choices are increasingly guided by infection control, patient comfort, and resource allocation. Therefore, this study aimed to evaluate the association between ICU room type (single vs. shared) and the occurrence of UE in mechanically ventilated patients.

## Materials and methods

Study design and setting

This was a retrospective observational cohort study conducted in the Emergency ICU of the Hospital de Clínicas, University of Campinas (UNICAMP), Brazil. Data were obtained from electronic medical records covering the period from March 2023 to March 2024.

The Emergency ICU is a mixed medical-surgical unit composed of both single-patient and shared rooms. Single rooms were enclosed by four walls, with one wall and the door predominantly made of glass, allowing visual access from the corridor. All ICU rooms, including both single and shared rooms, were connected to a centralized electronic monitoring system located at the nursing station, enabling continuous real-time surveillance of vital signs and alarms.

Study population and selection criteria

All patients aged ≥16 years admitted to the Emergency ICU during the study period and requiring invasive mechanical ventilation were eligible for inclusion. Pregnant patients were excluded. All eligible patients meeting these criteria during the study period were included. For patients with more than one ICU admission during the same hospitalization, only the first admission was considered. Patients with incomplete or missing key data were excluded from the analysis.

Patients were classified according to the type of ICU room (single or shared) in which the extubation event occurred. Although transfers between room types could occur during the ICU stay, extubation outcomes were attributed to the room configuration at the time of extubation.

Data collection and variables

The following variables were extracted from electronic medical records: demographic and clinical characteristics (age, sex, and comorbidities), severity of illness assessed by the Simplified Acute Physiology Score 3 (SAPS 3), ICU room type (single or shared), extubation type (planned or unplanned), occurrence of endotracheal tube displacement, extubation failure (defined as reintubation within 48 hours), ventilator-associated pneumonia (VAP), and mortality outcomes (ICU mortality, hospital mortality, and 30-day mortality).

Standard ICU sedation and delirium prevention protocols were applied according to institutional practice. However, detailed data on sedation regimens, sedation depth scores (e.g., RASS), delirium assessments (e.g., Confusion Assessment Method ICU), use of physical restraints, and nurse-to-patient ratios were not consistently available in the electronic records and therefore could not be reliably captured for analysis.

Definitions

VAP was defined as the appearance of a new or progressive pulmonary infiltrate occurring ≥48 hours after initiation of mechanical ventilation, accompanied by clinical signs of infection and microbiological confirmation, in accordance with established criteria.

UE included both self-extubation and accidental extubation. Extubation failure was defined as the need for reintubation within 48 hours following extubation.

Statistical analysis

Continuous variables were expressed as mean ± standard deviation or median with interquartile range, according to data distribution. Categorical variables were presented as absolute frequencies and percentages. Normality was assessed using the Shapiro-Wilk test. Comparisons between groups were performed using Pearson’s chi-square test or Fisher’s exact test for categorical variables, the Mann-Whitney U test for non-normally distributed continuous variables, and the independent-samples t-test for normally distributed continuous variables.

The primary analysis evaluated the association between ICU room type and extubation type (planned vs. unplanned). Secondary analyses examined associations between room type and VAP, endotracheal tube displacement, and mortality outcomes. A two-sided p value of <0.05 was considered statistically significant. Given the retrospective nature of the study, no a priori sample size or power calculation was performed.

Ethical considerations

The study was conducted in accordance with the Declaration of Helsinki and was approved by the Research Ethics Committee of the UNICAMP, Brazil (CAAE: 86937125.2.0000.5404). The requirement for informed consent was waived due to the retrospective design and the use of anonymized data.

## Results

Study population and baseline characteristics

During the study period, 118 patients aged ≥16 years who required invasive mechanical ventilation were included in the analysis. Of these, 45 patients (38.1%) were accommodated in single rooms and 73 (61.9%) in shared rooms, which contained four to six beds each.

The overall cohort had a mean age of 56.8 years and was predominantly male patients (54.3%). The mean SAPS 3 score was 76.4, indicating a critically ill population. Baseline demographic and clinical characteristics, including age, sex, weight, height, and SAPS 3 score, were comparable between patients admitted to single and shared rooms, with no statistically significant differences observed (Table 1).

**Table 1:** Epidemiological characteristics of patients admitted to the ICU according to the type of room There were no statistically significant differences between the groups SAPS3: Simplified Acute Physiology Score 3; ICU: intensive care unit

Parameter	Age (average), years	Weight (average), kg	Height (average), cm	SAPS 3 (average)
Single room	56.11	74.86	170.24	75.91
n	45	42	29	44
Error deviation	18.21	15.06	4.42	16.84
Median	61	77.50	170	74
% of total sum	37.7	38.5	36.3	38.2
% of total n	38.1	36.2	35.4	37.6
Shared room	57.27	74.39	170.17	76.70
n	73	67	52	72
Error deviation	19.62	16.87	5.38	16.32
Median	61	75	170	75.50
% of total sum	62.3	61.5	63.7	61.8
% of total n	61.9	63.8	64.6	62.4
Total	56.83	74.64	169.86	76.37
n	118	109	81	117
Error deviation	19.11	16.45	4.96	16.54
Median	61	75	170	75.50
% of total sum	100	100	100	100
% of total n	100%	100%	100%	100%

Extubation characteristics

Most extubations were planned and performed by the medical team (102/118; 86.4%). UE occurred in 16 patients (13.6%), including 14 cases of self-extubation (11.9%) and two cases of accidental extubation (1.7%).

A statistically significant association was observed between ICU room type and extubation type. UE was more frequent among patients in single rooms (10/45; 22.2%) than in shared rooms (6/73; 8.2%) (χ²(1) = 4.66; p = 0.031) (Table 2).

**Table 2:** Association between room type and clinical outcomes χ² = Pearson’s chi-square test; statistical significance set at p < 0.05 ICU: intensive care unit

Variable	Single room, n (%)	Shared room, n (%)	χ² (df)	p value
Extubation type				
Planned by the team	35 (77.8)	67 (91.8)	4.66 (1)	0.031
Unplanned (self/accidental)	10 (22.2)	6 (8.2)	-	-
Mortality				
Hospital mortality	14 (31.1)	31 (42.5)	1.52 (1)	0.217
ICU mortality	8 (17.8)	16 (21.9)	0.29 (1)	0.587
Ventilator-associated pneumonia	12 (26.7)	19 (26.0)	0.01 (1)	0.939
Endotracheal tube displacement	14 (31.1)	28 (38.4)	0.64 (1)	0.425

Ventilator-associated pneumonia

VAP occurred in 31 patients (26.3%). The incidence of VAP was similar between patients in single rooms (12/45; 26.7%) and shared rooms (19/73; 26%), with no statistically significant difference observed (p = 0.939) (Table 2).

Tube displacement and extubation failure

Endotracheal tube displacement was documented in 42 patients (35.6%). Tube displacement occurred more frequently among patients who experienced UE (10/16; 62.5%) than among those with planned extubation (32/102; 31.3%), showing a statistically significant association (χ²(1) = 5.85; p = 0.016) (Table 3).

**Table 3:** Association between extubation type and secondary outcomes χ² = Pearson’s chi-square test; statistical significance set at p < 0.05

Variable	Planned extubation, n (%)	Unplanned extubation, n (%)	χ² (df)	p value
Extubation failure ≤48 hours	19 (18.6)	5 (31.2)	1.36 (1)	0.244
Tube displacement	32 (31.3)	10 (62.5)	5.85 (1)	0.016

Extubation failure, defined as reintubation within 48 hours, was identified in 24 patients (20.3%). The proportion of extubation failure was numerically higher after UE (5/16; 31.2%) than after planned extubation (19/102; 18.6%), although this difference did not reach statistical significance (χ²(1) = 1.36; p = 0.244).

Mortality outcomes

Overall ICU mortality was 20.3% (24/118), and hospital mortality was 38.1% (45/118). No statistically significant association was observed between ICU room type and ICU mortality (p = 0.587) or hospital mortality (p = 0.217) (Table 2).

Patients who experienced extubation failure had significantly higher mortality rates. ICU mortality was 37.5% (9/24) in patients with extubation failure compared with 15.9% (15/94) in those without failure (p = 0.019). Hospital mortality was 62.5% (15/24) among patients with extubation failure vs. 31.9% (30/94) among those without failure (p = 0.006) (Table 4).

**Table 4:** Association between extubation failure and mortality χ² = Pearson’s chi-square test; statistical significance set at p < 0.05 ICU: intensive care unit

Outcome	Extubation failure, n (%)		χ² (df)	p value
	Yes	No		
ICU mortality	9 (37.5)	15 (15.9)	5.48 (1)	0.019
Hospital mortality	15 (62.5)	30 (31.9)	7.58 (1)	0.006

Additional outcomes and data availability

Data on ICU length of stay, hospital length of stay, duration of mechanical ventilation, and specific causes of UE were not consistently available in the electronic medical records and, therefore, could not be reliably analyzed or reported in the Results section.

## Discussion

This study evaluated the association between ICU room configuration and the occurrence of UE in a cohort of mechanically ventilated patients admitted to an emergency ICU. The population analyzed was critically ill, as reflected by a high mean SAPS 3 score and substantial ICU and hospital mortality, and showed clinical characteristics comparable to those reported in national and international cohorts of ventilated ICU patients [10-13]. These similarities support the external validity of the present findings.

A statistically significant association was observed between room type and extubation pattern, with a higher proportion of UEs occurring in single rooms. Importantly, this association was not accompanied by differences in ICU mortality, hospital mortality, or VAP. These findings suggest that ICU room configuration may influence surveillance-sensitive events, such as UE, without directly affecting major clinical outcomes. Given the retrospective observational design and the limited number of UEs, these results should be interpreted as associative rather than causal.

Previous investigations have described potential advantages of single-occupancy ICU rooms, including reduced environmental noise, improved sleep quality, and lower incidence of delirium, factors that may reduce agitation and device manipulation [14-17]. Conversely, reduced direct visual monitoring in single rooms has been associated with delayed recognition of adverse events, including self-extubation [14-17]. The present findings are consistent with this dual-effect hypothesis, in which reduced stimulation and reduced surveillance may coexist. This interaction may explain the predominance of planned extubations overall, alongside a proportionally higher frequency of UEs in single-room settings, without measurable impact on mortality or infection rates.

An additional relevant finding was the association between endotracheal tube displacement and UE. Tube displacement was significantly more frequent among patients who experienced UE, supporting its role as an early indicator of airway instability. Prior studies have identified inadequate fixation, shallow tube positioning, and patient mobilization as factors contributing to displacement and self-extubation [18,19]. Although causality cannot be established, the consistent association observed in this and other cohorts suggests that tube displacement may represent a clinically observable warning sign preceding unplanned airway loss.

Extubation failure occurred in approximately one-fifth of patients and was strongly associated with increased ICU and hospital mortality, regardless of room configuration or extubation type. Although the rate of extubation failure was numerically higher following UE, this difference did not reach statistical significance, likely reflecting limited statistical power. These findings align with previous studies demonstrating that reintubation within 48 hours is a robust prognostic marker associated with adverse outcomes, including increased mortality, prolonged ventilation, and extended hospitalization [20,21].

The incidence of VAP was similar between single and shared rooms, reinforcing the concept that prevention of this complication depends primarily on care processes rather than ICU architectural design alone [22-24]. This observation supports the view that ICU layout may differentially affect process-related events, such as surveillance and device safety, while infection-related outcomes remain more closely linked to adherence to established preventive practices.

Several limitations should be acknowledged. This was a single-center retrospective study, subject to information bias and variability in clinical documentation. Room classification was based on the location at the time of extubation, limiting assessment of cumulative environmental exposure. Important potential confounders, such as sedation depth, delirium assessments, use of restraints, staffing ratios, and direct visibility from nursing stations, were not uniformly available and could not be included in the analysis, as highlighted in prior studies addressing ICU organization and monitoring [25-27]. Additionally, the relatively small number of UE events limited statistical power for secondary analyses and precluded causal inference.

Previous studies have also shown that patients at higher risk of extubation failure may benefit from structured postextubation support strategies; however, the present study was not designed to evaluate the impact of such interventions [28,29]. Future investigations incorporating standardized clinical, organizational, and architectural variables are needed to further clarify the relationship between ICU design, monitoring practices, and extubation safety [30].

## Conclusions

This study indicates that the physical ICU environment may influence extubation patterns, with a higher proportion of UEs observed in single rooms, while no direct association was found with mortality, VAP, or other major clinical outcomes. The association between endotracheal tube displacement and UE supports displacement as a potential early marker of airway instability. In addition, extubation failure remained strongly associated with increased mortality, reinforcing its relevance as a prognostic indicator, independent of room configuration.

Overall, these findings suggest that while single-room ICU designs may offer advantages related to reduced environmental stress and patient comfort, they also highlight the importance of adequate surveillance to ensure patient safety. The results emphasize that major clinical outcomes in mechanically ventilated patients appear to be more closely related to care processes and patient severity than to architectural design alone.
